# Feasibility and constraints of Bragg peak FLASH proton therapy treatment planning

**DOI:** 10.3389/fonc.2024.1369065

**Published:** 2024-04-26

**Authors:** Nathalie Lövgren, Ingrid Fagerström Kristensen, Kristoffer Petersson

**Affiliations:** ^1^ Department of Oncology, Oxford Institute for Radiation Oncology, University of Oxford, Oxford, United Kingdom; ^2^ Clinical Oncology, Department of Clinical Sciences Lund, Lund University, Lund, Sweden; ^3^ Department of Haematology, Oncology and Radiation Physics, Skåne University Hospital, Lund, Sweden

**Keywords:** FLASH radiotherapy, proton therapy, feasibility studies, intensity-modulated proton therapy, Bragg peak

## Abstract

**Introduction:**

FLASH proton therapy (FLASH-PT) requires ultra-high dose rate (≥ 40 Gy/s) protons to be delivered in a short timescale whilst conforming to a patient-specific target. This study investigates the feasibility and constraints of Bragg peak FLASH-PT treatment planning, and compares the *in silico* results produced to plans for intensity modulated proton therapy (IMPT).

**Materials and method:**

Bragg peak FLASH-PT and IMPT treatment plans were generated for bone (n=3), brain (n=3), and lung (n=4) targets using the MIROpt research treatment planning system and the Conformal FLASH library developed by Applications SA from the open-source version of UCLouvain. FLASH-PT beams were simulated using monoenergetic spot-scanned protons traversing through a conformal energy modulator, a range shifter, and an aperture. A dose rate constraint of ≥ 40 Gy/s was included in each FLASH-PT plan optimisation.

**Results:**

Space limitations in the FLASH-PT adapted beam nozzle imposed a maximum target width constraint, excluding 4 cases from the study. FLASH-PT plans did not satisfy the imposed target dose constraints (D_95%_ ≥ 95% and D_2%_≤ 105%) but achieved clinically acceptable doses to organs at risk (OARs). IMPT plans adhered to all target and OAR dose constraints. FLASH-PT plans showed a reduction in both target homogeneity (p *<* 0.001) and dose conformity (non-significant) compared to IMPT.

**Conclusion:**

Without accounting for a sparing effect, IMPT plans were superior in target coverage, dose conformity, target homogeneity, and OAR sparing compared to FLASH-PT. Further research is warranted in treatment planning optimisation and beam delivery for clinical implementation of Bragg peak FLASH-PT.

## 1 Introduction

FLASH radiotherapy (FLASH-RT) is an emerging technique aiming to deliver ultra-high dose rate (≥ 40 Gy/s) radiation to treat patients. Pre-clinically, the use of FLASH-RT has been shown to induce a normal tissue sparing effect whilst maintaining the anti-tumour effectiveness of conventional radiotherapy techniques, known as the “FLASH effect” (1–14). Compared to conventional radiotherapy, the use of ultra-high dose rates for FLASH-RT allows for a substantial reduction in overall treatment times. FLASH-RT may therefore be able to mitigate uncertainties associated with intra-fractional motion, whilst also potentially increasing the availability and accessibility of radiotherapy treatments. These aspects of FLASH-RT could benefit treatments of targets where organ motion is a prevalent issue, normally requiring the use of large margins, gating, or tracking, such as the lungs, bowels, and liver (15). Treatments of regions where critical structures are at risk of receiving increased doses due to their proximity to the target, such as the brain and spinal cord, could also benefit from the normal tissue sparing of FLASH-RT.

Dose rates can be defined in different ways; two common definitions include the average dose rate (ADR) which accounts for the dead-time between each spot delivered, and the dose-averaged dose rate (DADR) which only considers the instantaneous dose rate delivered (16). The ‘dose rate coverage’ or ‘FLASH coverage’ are terms used to quantify the volume of an irradiated structure receiving dose at ultra-high dose rates. It is defined as the ratio of the organ at risk (OAR) volume receiving doses at ultra-high dose rates to the OAR volume receiving a defined threshold dose. Several studies report to achieve a FLASH dose rate coverage of approximately 80% to OARs when evaluating the ADR, however, this is highly dependent on how the study has defined ‘FLASH dose rate coverage’ as dose and dose rate thresholds vary (17–22)

Due to limited depth-penetration of clinically used electron beams, and vast power requirements and heating effects of producing ultra-high dose rate photons, protons are one of the most promising candidates for treatments of deep-seated targets. Heavier ion-beams and very-high energy electrons may also be contenders; however, ultra-high dose rate protons are readily available given few modifications to clinical delivery systems (23, 24). FLASH proton therapy (FLASH-PT) has therefore become a rapidly progressing branch within FLASH-RT research. Following the successful completion of the FAST-01 clinical trial which demonstrated the safe delivery of transmission beam FLASH-PT to patients, the FAST-02 trial is currently investigating the toxicities and pain relief associated with the treatment technique (25–27).

Despite transmission beam FLASH-PT showing promising results, the technique does not maximise the benefits associated with the use of the Bragg peak in standard proton therapy. Bragg peak proton therapy allows for improved dose conformity, with the use of fewer beams, and reduced doses to OARs when compared to transmission beam proton therapy (20, 28). The use of fewer beams allows for an increase in dose rates delivered by each beam, making Bragg peak FLASH-PT more favourable than transmission beam FLASH-PT. However, without accounting for a sparing effect, previous Bragg peak and transmission beam FLASH-PT treatment planning studies have shown inferiority to conventional proton therapy plans (20, 29). This is linked to both the beam delivery and treatment planning process. As the incorporation of dose rates into the plan optimisation has not been needed prior to FLASH-RT, the software capabilities and limitations are not well understood.

With regards to beam delivery, conventional energy-modulation techniques for standard Bragg peak proton therapy are not fast enough for ultra-high dose rates to be achieved. Instead, Bragg peak FLASH-PT can make use of patient-specific static energy degrades. Previous studies have adopted the use of range shifters or ridge filters, with the inclusion of collimators, along the path of a mono-energetic proton beam (20–22, 30). However, these systems have been associated with enlarged spot sizes due to multiple Coulomb scattering, and an increase in spot-to-spot distance to reduce overlap in the ridge filters, when compared to conventional proton therapy beam delivery (20–22, 29). How these increases impact the FLASH-PT treatments have not been well established; alternative beam setups and treatment planning optimisations could reduce their importance and thereby the deviations of FLASH-PT treatment planning from that of standard proton therapy. In addition to the difference in spot sizes, the time scale of gantry rotation for standard proton therapy machines are drastically longer than the treatment delivery time for FLASH-PT. Multi-beam fractions can therefore not be utilised for the ultra-high dose rate technique. To bypass this limitation, and to simplify the treatment setup due to the use of patient-and field-specific static energy degraders, it may be beneficial to deliver Bragg peak FLASH-PT using one beam per fraction.

The rapid progression of FLASH-PT, from pre-clinical studies to clinical trials, necessitates a full understanding of the capabilities, limitations, and safety of the treatment modality before it is implemented into clinical practice. Few studies focusing on treatment plans for FLASH-PT delivered in one beam per fraction have been conducted for Bragg peak proton beams, and no guidelines regarding the treatment planning process for this technique exists. The aim of this study was to investigate the feasibility and constraints of Bragg peak FLASH-PT treatment planning, using a novel beam setup and one beam per fraction, and determine if these plans are comparable to clinical intensity-modulated proton therapy (IMPT) plans.

## 2 Materials and methods

Ten patient cases were planned with IMPT and FLASH-PT and consisted of bone (n = 3), brain (n = 4), and lung (n = 3) targets. Target sizes ranged between 1.42-324.09 cm^3^, with a median size of 16.18 cm^3^. Details of the target types, defined OARs, target sizes, prescribed doses, and number of fractions for the individual patient cases are shown in **Table 1**.

**Table 1:** Table outlining the target type, defined organs at risk, target size, prescribed dose (Gy), and number of fractions used in the treatment plans for each patient case.

Patient Case	Target Type	Defined Organs at Risk	Target Size (cm^3^)	Prescribed Dose (Gy)	Number of Fractions
1	Bone	Body	62.20	8	1
2	Bone	Body	324.09	8	1
3	Bone	Left Lung, Right Lung, Body	207.07	8	1
4	Brain	Body	19.71	20	2
5	Brain	Brain, Body	1.42	30	3
6	Brain	Brain, Body	2.26	30	3
7	Brain	Brain, Body	12.65	34	2
8	Lung	Left Lung, Thoracic Wall, Body	11.98	45	3,4,5
9	Lung	Left Lung, Bronchial Tree, Body	10.66	45	3,4,5
10	Lung	Right Lung, Thoracic Wall, Great Vessel, Heart, Body	21.61	45	3,4,5

Treatment plans were produced using a MATLAB-based simulation software, utilising an open-source Monte Carlo engine known as MCsquare (many-core Monte Carlo) (31, 32). An open-source robust IMPT research treatment planning system (TPS), openMIROpt, produced by UCLouvain and accessed through OpenReggui was used to produce the IMPT plans (33). FLASH-PT plans were generated using the same open-source software, along with a ConformalFLASH library (www.openFLASH.software), developed by Ion Beam Applications SA (IBA), allowing for dose rate calculation and optimisation (34). The treatment plans were simulated for use on the IBA Proteus^®^Plus (Louvain-la-Neuve, Belgium) machine. The maximum field-size currently available from IBA for this beam setup is 8×8×8 cm^3^. The IMPT spots were placed in a hexagonal pattern for optimal dose coverage, and a constant relative biological effectiveness (RBE) of 1.1 was assumed. Robust planning is not yet available for ConformalFLASH, and so planning target volume-based treatment planning was adopted for all treatment plans to allow for a fair comparison. Additionally, a couch angle of 0^°^ was used as the research TPS does not allow for uses of other angles. Proton beams were planned using a cyclotron with a clinically used beam current of 300 nA for IMPT, and the maximum available beam current of 500 nA for FLASH-PT.

FLASH-PT beams were simulated using monoenergetic spot-scanned protons at an energy of 230 MeV. The ultra-high dose rate protons traverse through a conformal energy modulator (CEM), a range shifter, and an aperture, before reaching the target volume. The CEM is a patient- and field-specific ‘hedgehog’, which modulates the protons to the 3D shape of the target; it is made from a 3D printed plastic-like material called ‘Tusk’. The range shifter is used to set the distal range of the delivered dose to the target, and the aperture to reduce dose contributions from scattered particles and to sharpen the penumbra.

Standard IMPT treatment plans are optimised using either single-field optimisation (SFO) or multi-field optimisation (MFO). Plans are optimised using SFO when a single beam is used to cover the entire target and each beam is optimised independently from the others. MFO allows for use of multiple beams to cover the entire target, and the dose contribution of each beam is considered in the dose calculation and optimisation of the other beams. This improves target coverage whilst minimising dose to surrounding structures, making MFO the standard optimisation technique used in treatment planning (29). As Bragg peak FLASH-PT requires dose delivery to be made using one beam per fraction, due to ultra-high dose rate requirements and to improve target homogeneity, all FLASH-PT plans in this study were optimised using SFO. For treatments of simpler structures, such as the vertebrae cases in **Table 1**, a single-field fraction was used. However, for more advanced structures, such as the brain and lung targets, the use of a single field would lead to increased doses to OARs and worsened dose conformity. In these cases, multiple single-field fractions were used. Different fraction numbers were investigated for the three lung cases, as seen in **Table 1**. Initially, the idea was to generate plans in line with standard fractionations schemes for this target type by using 3 and 5 fractions. However, with an increase in number of fractions there is a decrease in intra-fraction dose, and thus dose rates, to OARs. To determine if there is a middle ground between reducing doses to OARs and increasing the dose rates delivered, the use of 4 fractions was also investigated. In addition to the standard MFO IMPT plans being generated, SFO IMPT plans were also produced to allow for fair comparisons of the SFO FLASH-PT plans to IMPT. This will show any discrepancies between the plans which are solely due to the use of ultra-high dose rates and those which may be due to the use of SFO instead of MFO. The sum of the SFO plans, for all fractions, will be used to compare the FLASH-PT and IMPT SFO plans to those of IMPT MFO.

A percentile dose rate definition, similar to that of the ADR, is adopted by the ConformalFLASH library; it is defined as the ratio of 98% of dose delivered to the time taken to deliver that dose (34). A minimum dose rate constraint of ≥ 40 Gy/s is implemented for each OAR volume receiving a dose ≥ 2 Gy. Dose rate coverage in this study is therefore defined as the volume of OAR which adheres to this constraint. As pre-clinical studies have found varying values for the dose modifying factor (DMF) for the FLASH effect, a DMF of 1 is used in all treatment plans (8, 10, 11). A “FLASH effect” is therefore not assumed in the FLASH-PT treatment plans. Each plan was normalised such that D_50%_ = D*_p_*, where D*_p_* is the prescribed dose. Dose constraints used for the target structures were D_95%_ ≥ 95% and D_2%_ ≤ 105% of the relative target volumes.

Dose distribution maps, dose volume histograms (DVHs), and dose statistics were used to qualitatively compare the dose distributions of IMPT MFO, IMPT SFO, and FLASH-PT plans. Quantitative comparisons are made using DVH parameters and dose statistics; this allows for conformity and homogeneity indices to be determined, along with statistical comparisons of the DVH parameters using the two-sided Wilcoxon Rank Sum Test, Spearman’s Rank Correlation Coefficient (*ρ*_SRC_), and Pearson Correlation Coefficient (*ρ*_PCC_), with significance defined for p *<* 0.05. Different samples involving the mean dose, V_95%_, V_100%_, V_105%_, V_107%_, D_2%_, D_5%_, D_95%_, and D_98%_ were tested. Clinical acceptability for doses delivered to OARs were defined using the European Particle Therapy Network (EPTN) consensus and the Quantitative Analysis of Normal Tissue Effects in the Clinic (QUANTEC) review (35, 36).

The radiation conformity index (RCI) was evaluated using the ratio of the target volume to the total volume receiving 95% of the prescribed dose (RCI = V*_target_*/V_95%_) (37). The homogeneity index (HI) was calculated using HI =(D_2%_-D_98%_)/D_50%_ as defined in the ICRU Report 83 (38). All treatment planning data was included in these comparisons.

## 3 Results

The field size currently available from IBA imposed a size constraint on the targets. This is due to limitation in the space between the CEM and range shifter in the FLASH-PT adapted beam nozzle. Treatment plans could therefore not be simulated for targets with width (inline or crossline) above 70 mm. Patient cases 1, 2, 3, and 4 all exceeded this size limit and were therefore excluded from the study. A minimum angle criterion of ≥ 40° between each beam was chosen to reduce beam overlap and hotspots for the SFO plans. However, the space limitation also affected which beam angles that could be used, likely due to the target sizes along with the margins for spot placements exceeding 70 mm. Three FLASH-PT plans could therefore not adhere to the minimum angle criterion: Case 8 (5 fractions) and Case 10 (3 and 5 fractions).

**Figure 1:**
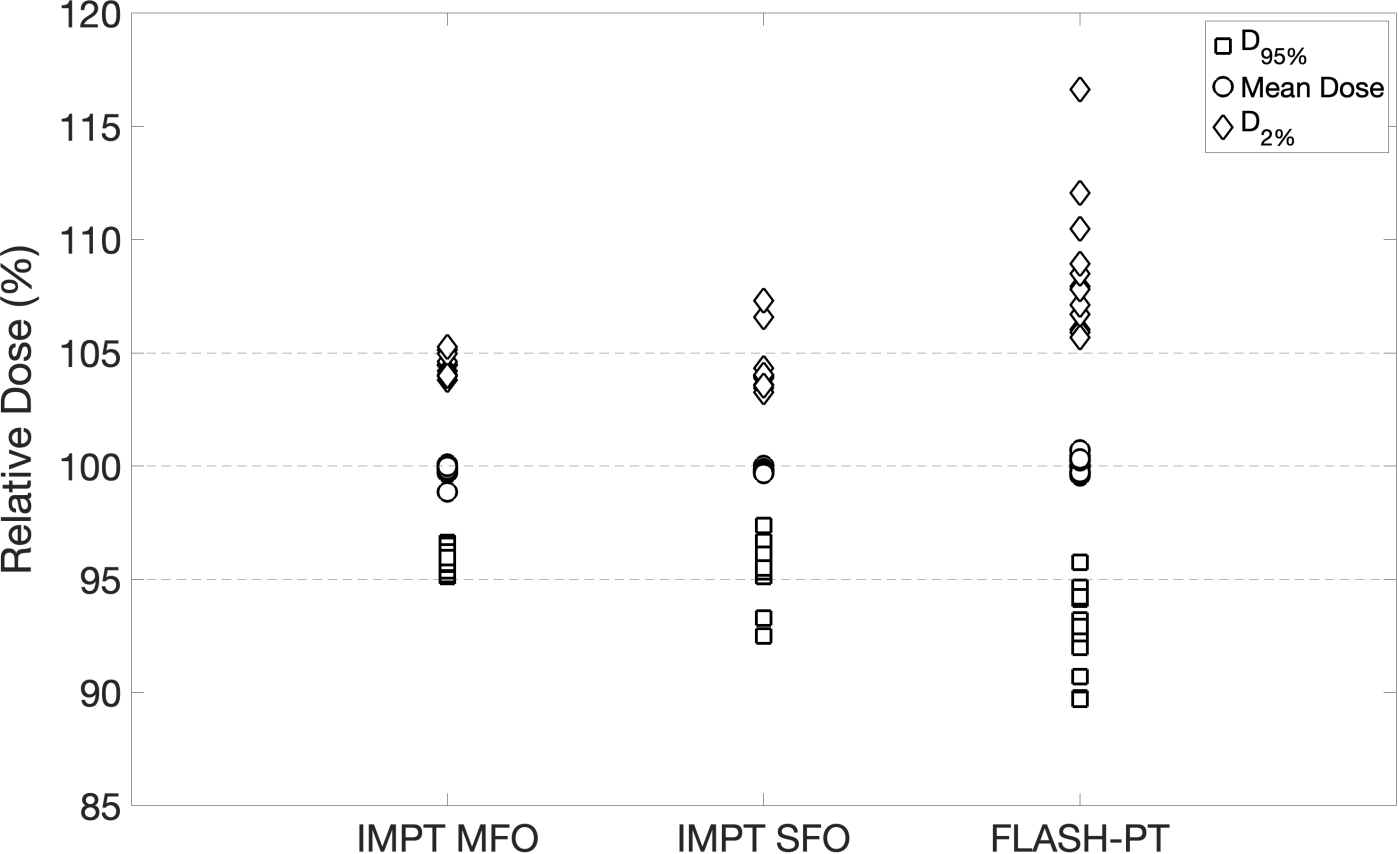
Plot summarising target dose metrics. D_95%_ (square), mean dose (circle), and D_2%_ (diamond) are normalised with respect to the prescribed dose for all patient cases and for each treatment technique. The dashed lines indicate 95%, 100%, and 105% of the prescribed dose.

**Figure 2:**
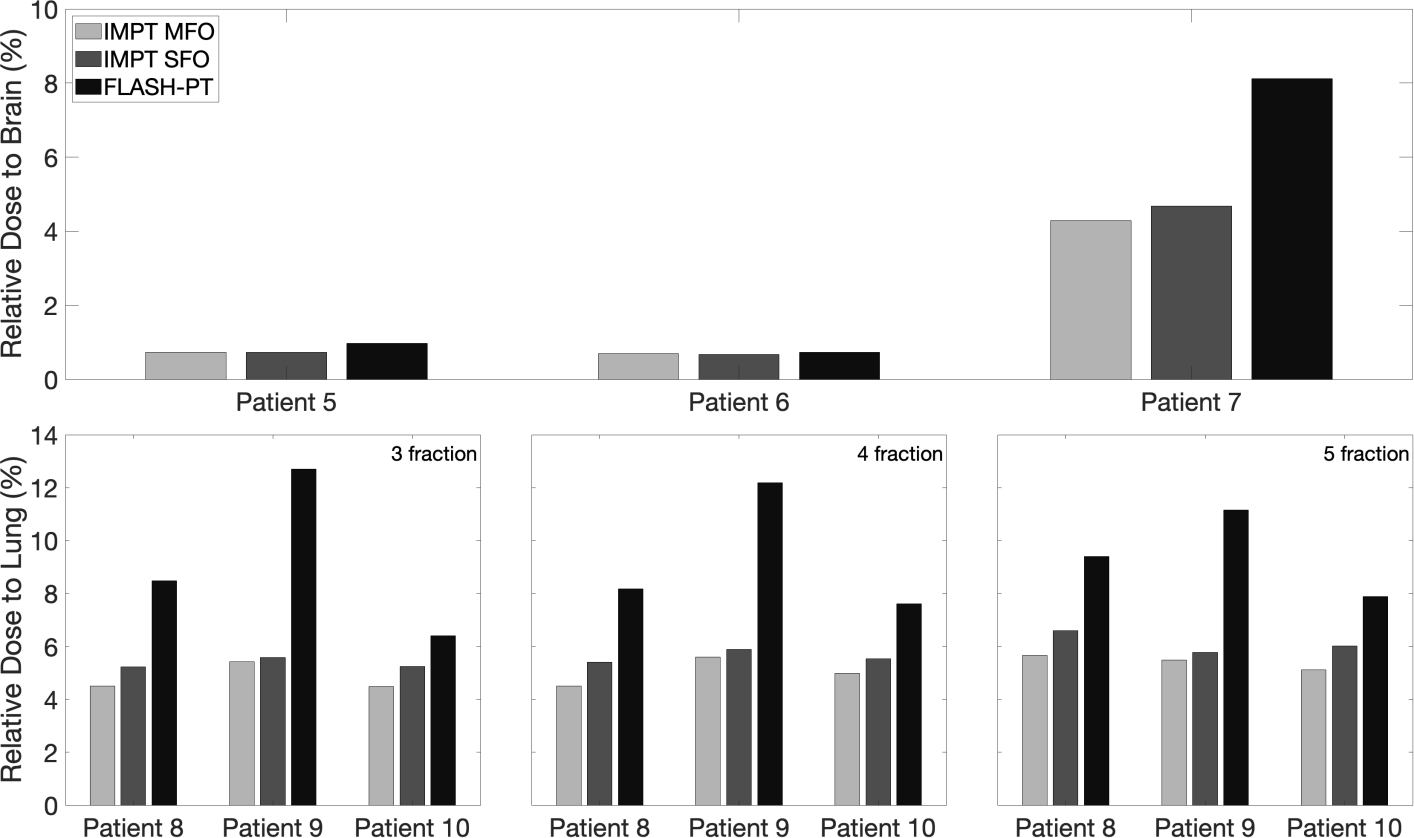
Bar charts representing the mean doses delivered to OARs for IMPT MFO, IMPT SFO and FLASH-PT, normalised with respect to the prescribed dose for each patient case. The considered OAR structures are the total brain (top) and the lung side in which the target is located (bottom). For the lung cases, the doses to the lung are shown for the 3-,4-, and 5-fraction plans.

**Figure 3:**
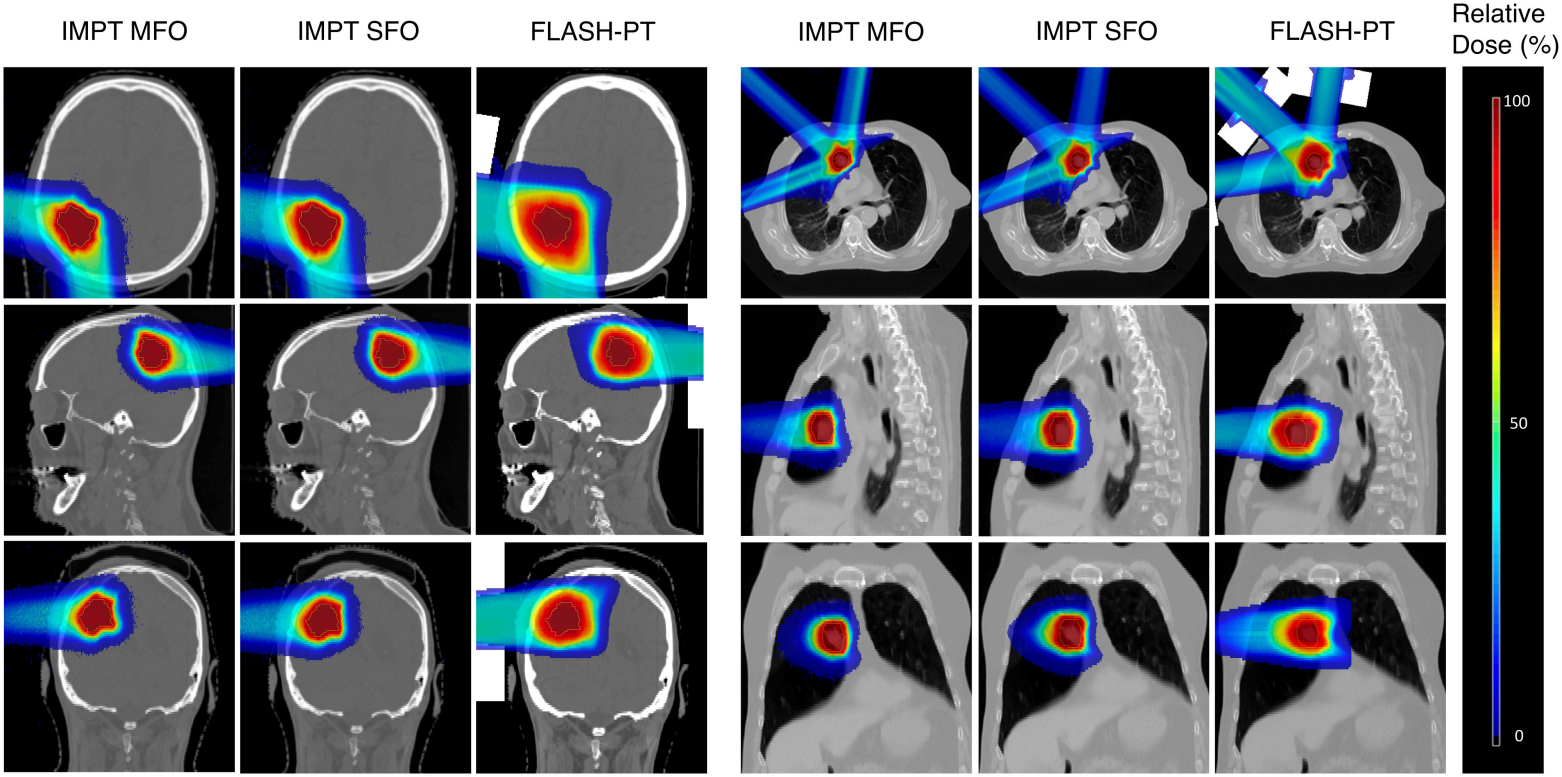
Dose maps for an example brain (Case 7) and lung case (Case 10, 3 fractions). Comparisons of IMPT MFO, IMPT SFO, and FLASH-PT are made in the axial, sagittal, and coronal directions. Doses are normalised with respect to the prescribed dose.

IMPT MFO achieves the clinical dose constraints imposed, with D_95%_ *>* 95% and D_2%_ *<* 105%, as seen in **Figure 1**. IMPT SFO has two cases where these dose limits are not achieved: Cases 5 and 6 which have the two smallest target volumes, as seen in **Table 1**. FLASH-PT, however, only achieves similar mean doses to the other two IMPT techniques, with D_95%_ and D_2%_ being significantly different to both IMPT MFO and IMPT SFO, as shown in **Table 2**. Since IMPT MFO and IMPT SFO are comparable, the discrepancy seen for FLASH-PT is not only due to the use of SFO but also due to the limitations imposed for the use of ultra-high dose rate protons. In addition to this, FLASH-PT is associated with a significant increase in dose to OARs, as seen in **Figure 2**. Nevertheless, the doses are still within clinically acceptable limits, as defined by the EPTN consensus and the QUANTEC review (35, 36). An increase in the dose at the distal edge of the targets were observed for all FLASH-PT plans compared to IMPT, with examples shown in **Figure 3**. This is likely due to range straggling of the 230 MeV protons following interactions with the energy degraders used in this beam setup; and would also contribute to the increase in dose to OARs observed for FLASH-PT. Dose volume histograms (DVHs) for the different cases, fractionation schemes, and treatment techniques are shown in the **Supplementary Appendix**.

**Table 2:** Table of median values of: mean dose (%), D_98%_ (%), D_95%_ (%), D_5%_ (%), D_2%_ (%), V_95%_ (%), V_100%_ (%), V_105%_ (%), V_107%_ (%), radiation conformity index, and homogeneity index for IMPT MFO, IMPT SFO, and FLASH-PT.

Dose Parameters	IMPT MFO	IMPT SFO	FLASH-PT
Mean Dose (%)	99.86	99.79	99.98
D_98%_ (%)	94.40	94.37	91.64 ^*^
D_95%_ (%)	95.79	95.54	93.06 ^**^
D_5%_ (%)	103.69	103.30	106.76 ^**^
D_2%_ (%)	104.47	103.98	107.87 ^***^
V_95%_ (%)	96.87	96.76	87.26 ^**^
V_100%_ (%)	49.99	50.46	50.24
V_105%_ (%)	0.89	0.41	10.95 ^***^
V_107%_ (%)	0.02	0.01	4.20 ^**^
Radiation Conformity Index (RCI)	0.93	0.87	0.80
Homogeneity Index (HI)	0.10	0.09	0.16 ^***^

The Wilcoxon Rank Sum Test was performed using the dose parameters for each patient case and for each treatment technique with the median value being used as a representative value. FLASH-PT plans are significantly inferior in the majority of dose metrics compared to both IMPT techniques. ^∗∗∗^p < 0.001, ^∗∗^p < 0.01, ^∗^p < 0.05.

Spearman’s Correlation Coefficient showed a very strong (p *<* 0.01) negative correlation (*ρ_SRC_*) = -0.80) between the RCI and the target size in the IMPT SFO case. The use of SFO therefore appears to reduce conformity, however, this result was not found for the Bragg peak FLASH-PT plans, likely due to the inclusion of the aperture in the beam setup. Pearson’s Correlation Coefficient showed a significant (p *<* 0.05) positive correlation (*ρ_PCC_*) = 0.63) between the HI and the target sizes for the FLASH-PT plans. An increase in target size is therefore related to a linear increase in HI, and thus worsened target homogeneity. This relationship was not found for IMPT MFO or IMPT SFO.

**Figure 4:**
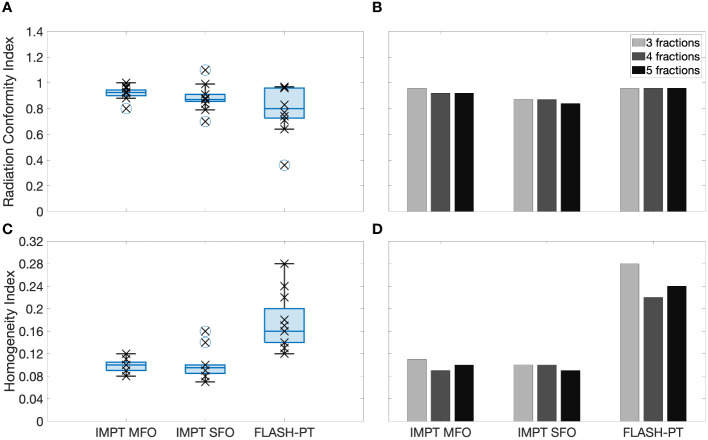
Left, boxplots of the **(A)** radiation conformity index (RCI) and **(C)** homogeneity index (HI) evaluated for each patient case and each treatment technique. Right, bar charts of the **(B)** RCI and **(D)** HI for 3,4, and 5 fractions shown for Case 10. Each point (x) shows the exact RCI and HI for each patient case. The line within the box represents the median value, the bottom and top of the box represents the 25*^th^* and 75*^th^* percentiles, the whiskers show the range of the data points (excluding outliers), and outliers are shown using individual (o) markers.

FLASH-PT plans appeared to be dependent on the target shape, position, smoothness of distal edge of the target, and distance from the nozzle of the beam to the target. Therefore, treatment plans generated using the same treatment planning parameters (spot spacings, layer spacings, and margins for spot placements) varied greatly with respect to the dose distributions and subsequently the plan quality achieved. To circumvent this, treatment planning parameters suitable for each patient case and each treatment fraction were used and obtained through manual parameter optimisation. Different treatment planning parameters were therefore required for each treatment fraction, with significantly larger spot spacings, layer spacings, and margins for spot placement needed to achieve similar mean doses and conformity to that of standard IMPT. Spot and layer spacings were at least twice as large for FLASH-PT plans compared to those used for IMPT. An increase in the spacings lead to a decrease in the number of spots placed on the target, making it more difficult for the TPS to generate and optimise conformal and homogeneous treatment plans (**Figures 4A, C**). Additionally, larger margins for spot placements improved the target coverage of the FLASH-PT plans, albeit at the expense of increasing the dose to nearby OARs. Details on the spot spacings, layer spacings, and margins of spot placements for each patient case and treatment fraction are shown in **Tables S1–S3** in the **Supplementary Appendix**.

FLASH-PT plans were less conformal than both IMPT plans (**Figures 3**, **4A**), although not significantly. Dose conformity for Bragg peak FLASH-PT can be improved by increasing the resolution at which the CEM is calculated, however, at the expense of drastically increasing the simulation time using this TPS. IMPT MFO and IMPT SFO plans produced comparable HI; this indicates that the inferior target homogeneity of FLASH-PT, seen in **Table 2** and **Figure 4C**, is mainly attributed to the limitations imposed for use of ultra-high dose rate protons. More specifically, the worsened target homogeneity is likely due to the increase in spot sizes and subsequent decrease in number of spots than can be placed, in addition to the impact of target size. Furthermore, as there are multiple objectives in the TPS, the optimiser appears to struggle to fulfil all criteria with fewer spot numbers than what is conventionally used for IMPT.

Case 10, having the largest target size and the largest number of OARs (**Table 1**), was selected as an example case to investigate the capabilities of the treatment planning optimiser and the subsequent treatment planning quality. The large target size, and the number of OARs, for this case led to inferior FLASH-PT treatment plans and so it is shown as a “worse-case” scenario in comparison to the rest of the samples. As shown in **Figure 4B**, the FLASH-PT RCI is comparable to both IMPT techniques, for all fractionation schemes. **Figure 4D**, however, shows a drastic reduction in target homogeneity for FLASH-PT when compared to IMPT (MFO and SFO). The 3-fraction FLASH-PT plan appeared to be the most inferior in terms of homogeneity; this is expected as reducing the number of beams will reduce the target homogeneity. This was also observed for the FLASH-PT plans generated for the other lung cases using 3 fractions. However, comparing DVHs shown in the **Supplementary Appendix (Figures S2–S4)**, increasing the number of fractions does not generally improve target coverage or OAR doses.

**Figure 5:**
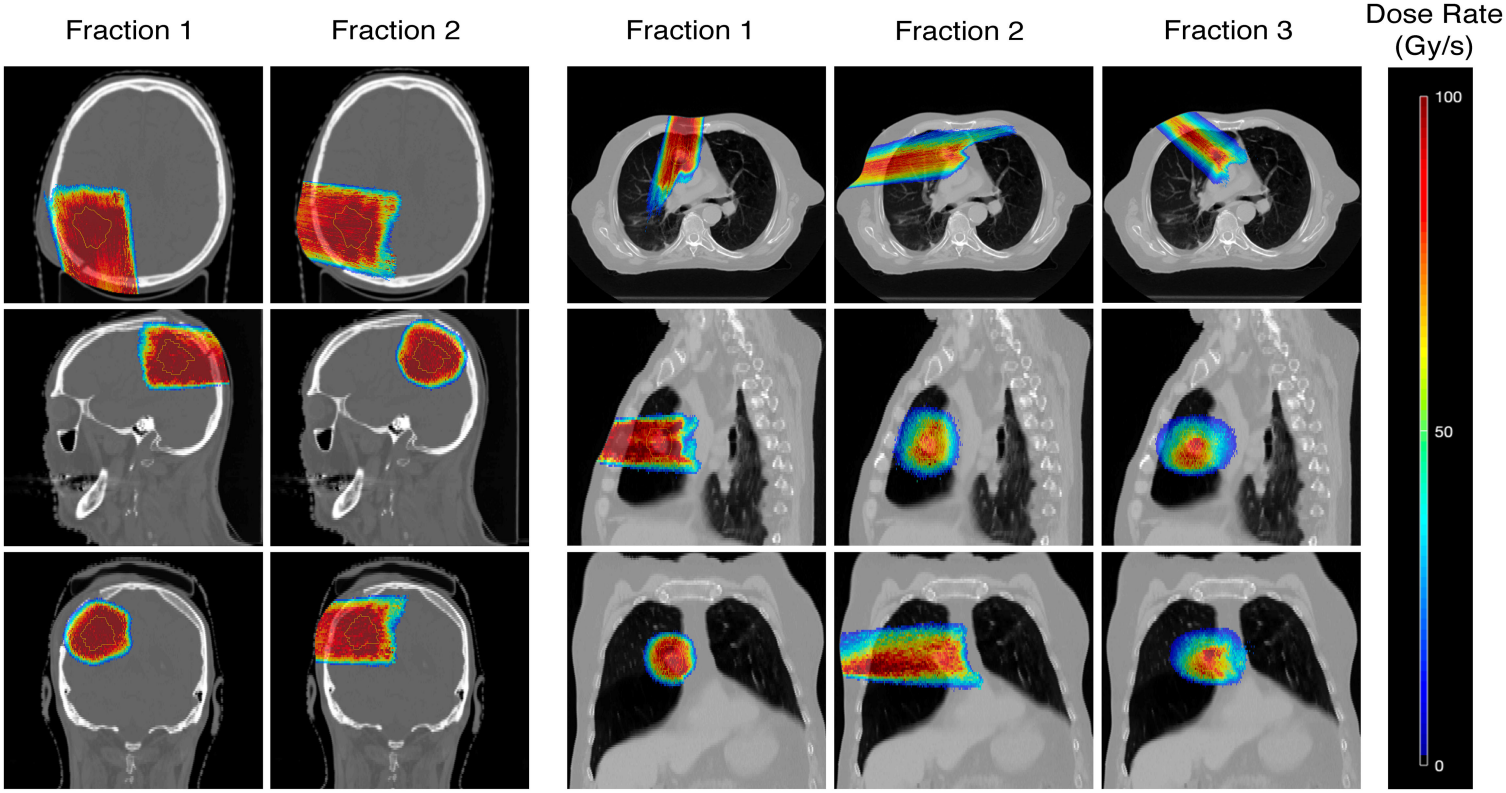
Dose rate maps for an example brain (Case 7) and lung case (Case 10, 3 fractions), showing the percentile dose rate in Gy/s. Comparisons of the dose rates achieved in each fraction are made in the axial, sagittal, and coronal directions.

**Figure 6:**
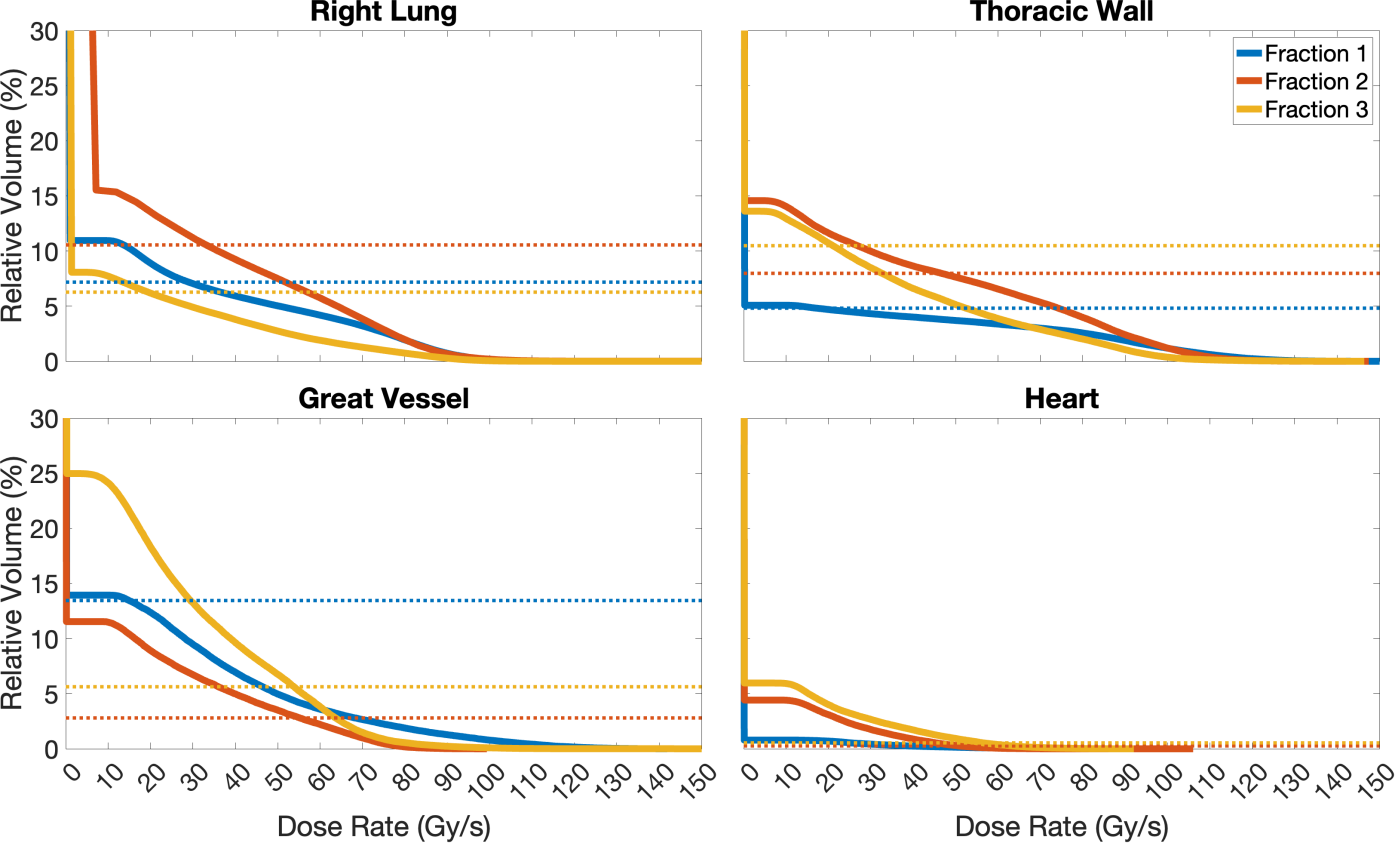
Dose rate volume histograms of the Right Lung, Thoracic Wall, Great Vessel, and Heart structures for Case 10, separated for the 3 fractions planned. For simplicity, as no OAR receives FLASH dose rates to relative volumes ≥ 30% in this study, only relative volumes in the range of 0-30% are included in these plots. Solid lines represent the dose rate, different colours indicate the different fractions planned, and dashed lines represent the volume of the OAR which receives doses ≥ 2 Gy (below line). In the case of the Right Lung, the optimisation criterion was not fully achieved, as there are volumes receiving doses ≥ 2 Gy (dashed lines) that do not receive the dose at dose rates ≥ 40 Gy/s (solid lines); fraction 2 nearly fulfils it. Similar, for the Thoracic Wall, only fraction 2 fully achieves the dose rate optimisation criterion, while fractions 1 and 3 do not. The criterion is fully achieved for fractions 2 and 3 for the Great Vessel, and for all fractions for the Heart.

For the same example case, variations in dose rates appeared to be dependent on the OARs as well as the number of fractions, and thus beams, used to deliver the treatment (**Figures 5**, **6**). Comparing the brain and lung case shown in **Figure 5**, the use of two fractions to treat a target with few density differences along the path of the beam (brain) generates higher dose rates to the surrounding normal tissues compared to the use of three fractions treating a target in a location where more density differences are present (lung). A further decrease in dose rates was also observed when increasing the number of fractions used for the lung cases.

Focusing more on the “worst-case” scenario (Case 10), the minimum dose rate constraint was not fully achieved for the Right Lung (for any fraction); this means that there were volumes receiving ≥ 2 Gy (dashed lines) that did not receive these doses at dose rates ≥ 40 Gy/s (solid lines), as shown in **Figure 6**. This is likely due to low-dose, and therefore dose rate, regions at the edges of the beam, as seen in **Figure 5** and is a consequence of strict OAR dose constraints for this structure. Similarly for the Thoracic Wall, only fraction 2 fully achieved the dose rate optimisation criterion, while fractions 1 and 3 did not. However, the criterion was fully achieved for fractions 2 and 3 for the Great Vessel and for all fractions for the Heart. Larger OARs, such as the Right Lung and Thoracic Wall will have more low-dose rate regions due to the beam-path chosen and beam-spread compared to structures such as the Heart and Great Vessel, which due to their proximity to the target receive most of the dose at much higher dose rates.

**Figure 7:**
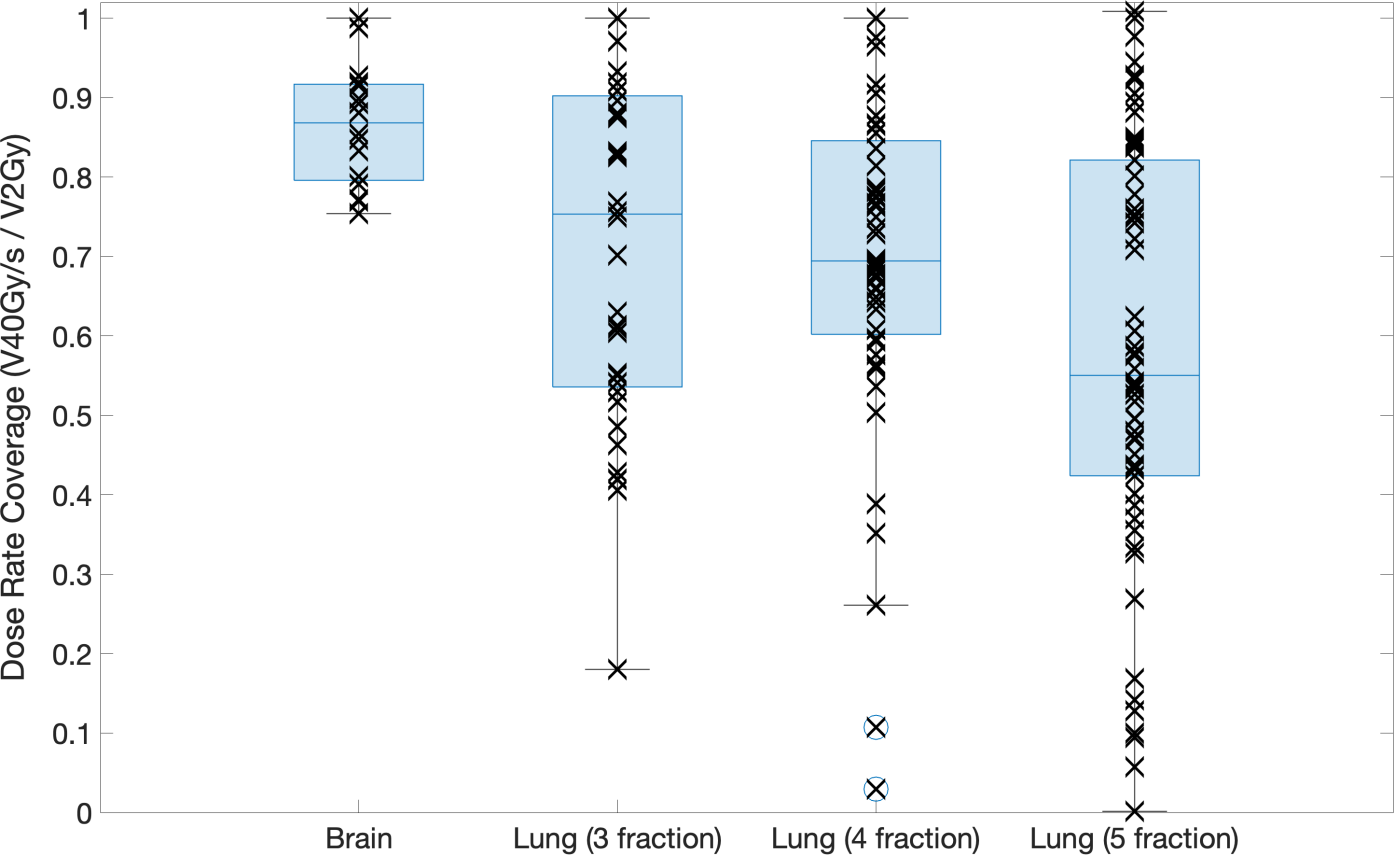
Boxplot showing the dose rate coverage of the OARs defined for the brain and lung cases for each fractionation scheme investigated. Each point shows the exact dose rate coverage for each OAR in each patient case, the line across the box represents the median value, the bottom and top of the box represent the 25*^th^* and 75*^th^* percentiles, the whiskers show the range of the data point (excluding outliers).

Dose rate maps and dose rate volume histograms (DRVHs) give a good representation of the intra-fraction dose rates achieved for a patient case, however, to allow for comparisons between target types and fraction numbers, dose rate coverages were evaluated using DRVH dose rate metrics and the corresponding DVH dose metrics. Median values of dose rate coverages for the brain plans and 3-, 4-, and 5- fraction lung plans are 87%, 71%, 69%, and 56%, respectively. For the different lung fractionation schemes investigated, the 5-fraction plans had significantly (p *<* 0.05) lower dose rates than the 3- and 4-fraction plans. This is likely due to the reduced intra-fraction dose making it harder for the optimiser to fulfil the ultra-high dose rate objective, as it paradoxically must increase the dose to achieve FLASH dose rates whilst also adhering to a maximum OAR dose constraint. Increasing the number of fractions lead to an increase in the spread of dose rate coverages achieved to OARs, as seen in **Figure 7**. This was expected as the dose per fraction decreased.

Significantly higher dose rate coverages were achieved for the brain cases compared to the lung cases for all fractionation schemes investigated. This could be due to the increase in density differences along the path of the beam for the lung cases compared to the brain cases.

## 4 Discussion

Due to the novelty of Bragg peak FLASH-PT, few feasibility studies focusing on the clinical implementation, and specifically the treatment planning process, have been conducted. The aim of this study was to determine this feasibility, and outline any constraints, of producing Bragg Peak FLASH-PT treatment plans delivered in one beam per fraction, and whether the generated plans were comparable to those for a clinically used technique such as IMPT. Without accounting for the sparing effect attributed to ultra-high dose rates, IMPT plans had better target coverage, dose conformity, target homogeneity, and OAR sparing compared to Bragg peak FLASH-PT using this beam setup and its implementation into the MIROpt TPS.

An interplay between the Bragg peak FLASH-PT beam setup, target location, fractionation scheme, and treatment planning optimisation is evident. Density differences along the path of the beam leads to variations in the treatment plans; target areas with few density differences, such as the brain, showed less variations whilst also achieving better target coverage, reduced doses to OARs, and improved dose rate coverage compared to the lung cases. In this study, brain cases appear to be more favourable than lung when using Bragg peak FLASH-PT, although the sample size is not large enough to ensure statistical significance.

The FLASH coverage for the brain and lung (3 fractions) cases, with OAR volumes receiving doses ≥ 2 Gy at dose rates ≥ 40 Gy/s, is comparable to the coverages of approximately 80% reported in previous treatment planning studies. The achieved FLASH coverage appears to be independent of patient case and target type when comparing brain and lung targets. In terms of fractionated treatment delivery, increasing the number of fractions for the lung cases did not result in any major differences in the target dose distributions or doses to OARs. A reduction in the intra-fraction dose rate coverage was found, as expected due to the intra-fraction dose being reduced. Fewer fractions will be more favourable if the FLASH sparing effect is to be fully exploited, however, ensuring clinically acceptable target coverage is still a priority.

The implementation of the CEM from the Conformal FLASH library to the MIROpt research TPS, the sample size, and the imposed target size constraint, are clear limitations in this study. However, in addition to being open-source and thereby very accessible, the use of the Conformal FLASH library in conjunction with MIROpt provided valuable information with regards to the treatment planning process of Bragg peak FLASH-PT. This includes the evaluation of fractionated beam delivery, dose comparisons between target types, the effect of target size on the planning quality, and the capability of achieving FLASH dose rates to the majority of the irradiated OAR volumes. Nevertheless, implementing the same beam setup into a more clinically used and validated system, such as RayStation^®^ (RaySearch AB, Stockholm, Sweden), will allow for more efficient optimisation with additional degrees of freedom. This should improve the Bragg peak FLASH-PT plan quality. In addition to this, further studies should include a greater sample size, potentially focus on target types of varying sizes, and explore the use of hypo-fractionated regimens for this novel technique.

## 5 Conclusion

The aim of this study was to determine the feasibility and constraint of producing IMPT equivalent Bragg Peak FLASH-PT treatment plans using an open-source TPS. Without accounting for the FLASH sparing effect attributed to delivery at ultra-high dose rates, IMPT plans were superior in target coverage, dose conformity, target homogeneity, and OAR sparing compared to Bragg peak FLASH-PT using this beam setup. Establishing whether the limitations found are software or hardware related will be key in further developing, improving, and demonstrating the feasibility of clinically implementing Bragg peak FLASH-PT.

## Data availability statement

The raw data supporting the conclusions of this article will be made available by the authors, without undue reservation.

## Ethics statement

EPN Lund 2013/742" allowed us the use of patient CT scans in radiotherapy research and development.

## Author contributions

NL: Writing – original draft. KP: Writing – review & editing, Supervision, Funding acquisition. IF: Writing – review & editing, Resources.
