# Supplementary material for: Feasibility and constraints of Bragg peak FLASH proton therapy treatment planning
Source: Front Oncol. 2024 Apr 26;14:1369065. doi: 10.3389/fonc.2024.1369065 (PMC11082391; doi:10.3389/fonc.2024.1369065)
Supplement: Supplementary file 1 [file DataSheet_1.pdf]

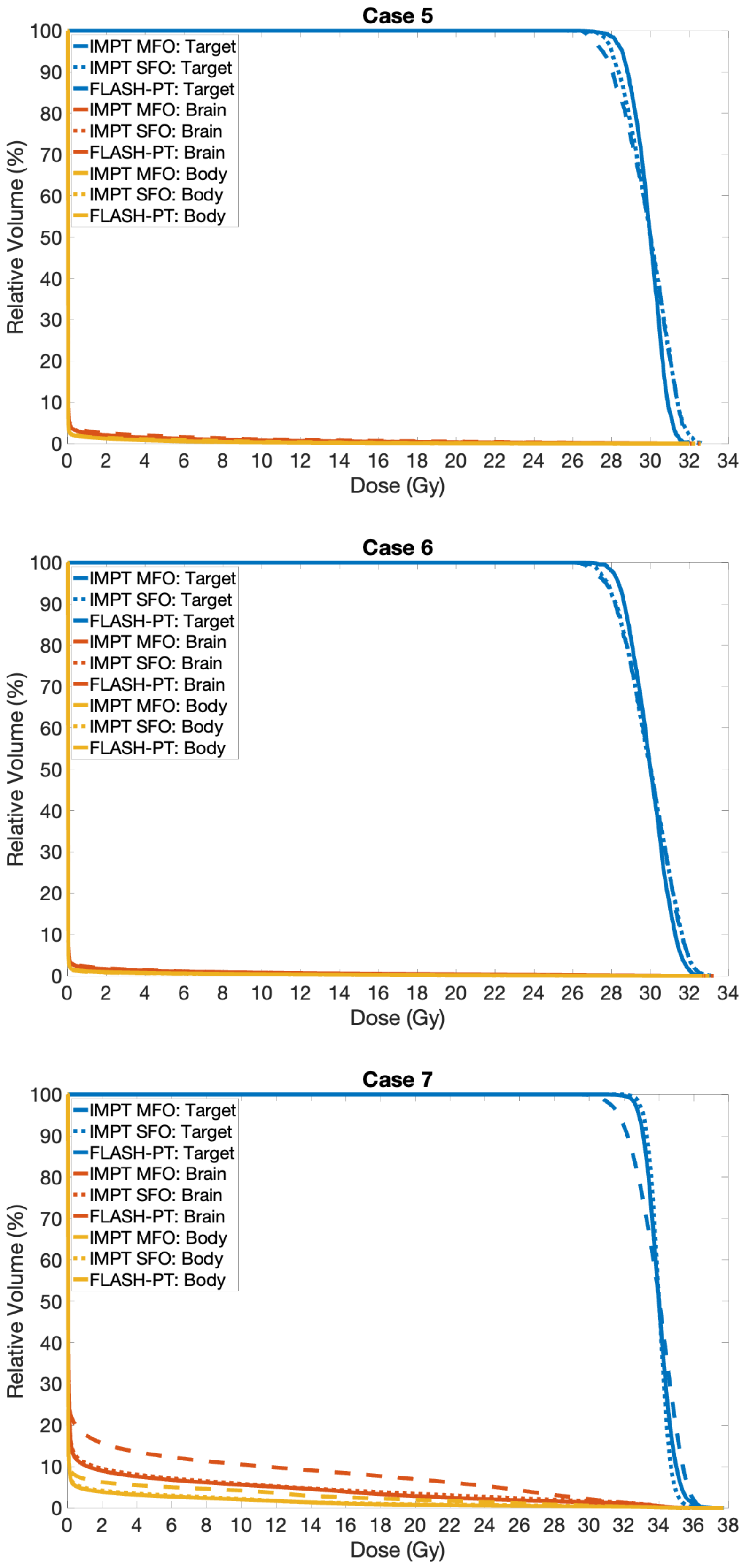

Figure S1: Dose volume histograms of the three brain cases investigated. In each case, doses to the target and OAR structures for each treatment technique (IMPT MFO, IMPT SFO, and FLASH-PT) are displayed.

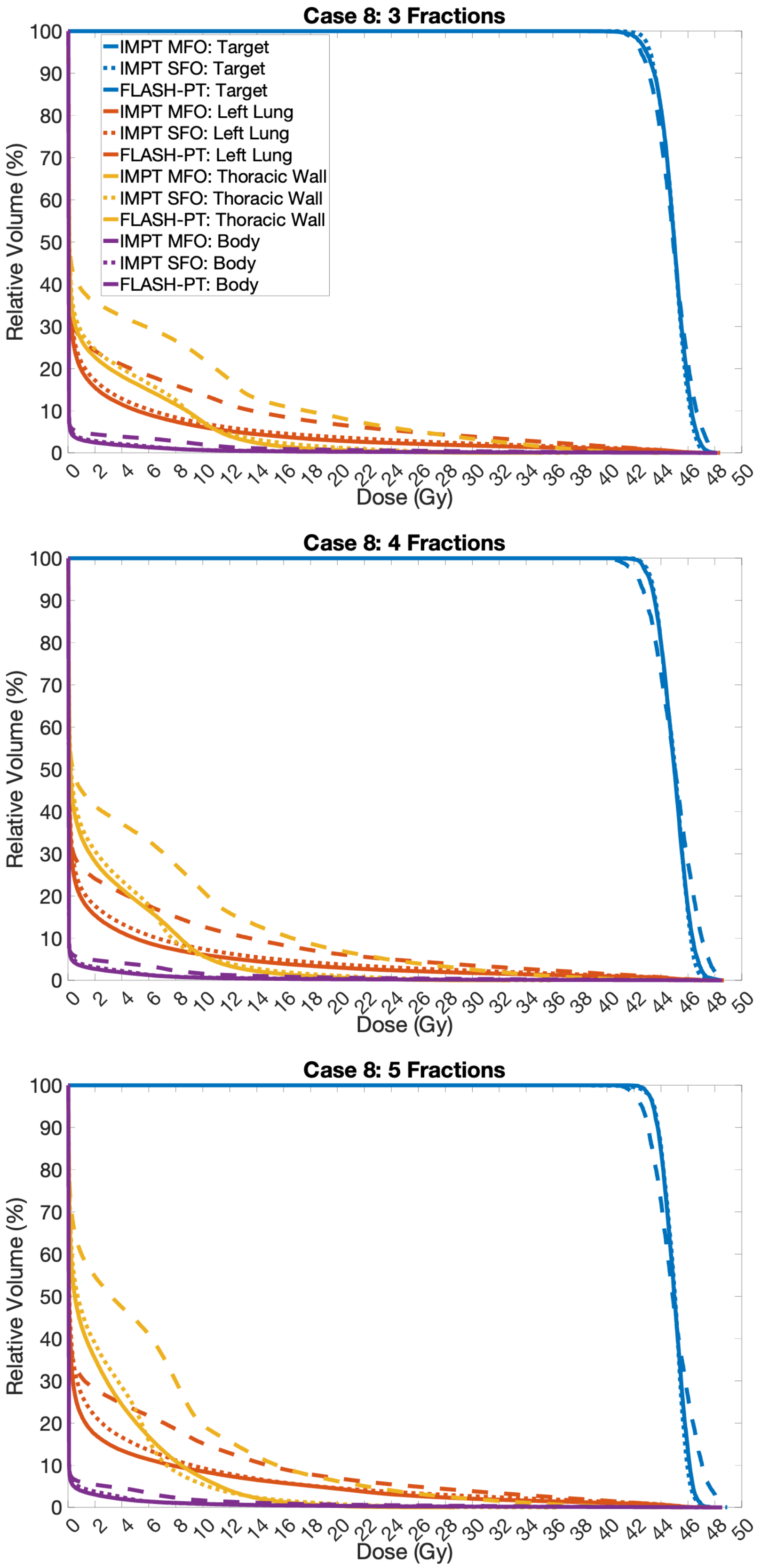

Figure S2: Dose volume histograms for Case 8. In each case, doses to the target and OAR structures for each treatment technique (IMPT MFO, IMPT SFO, and FLASH-PT), and for each fractionation scheme, are displayed.

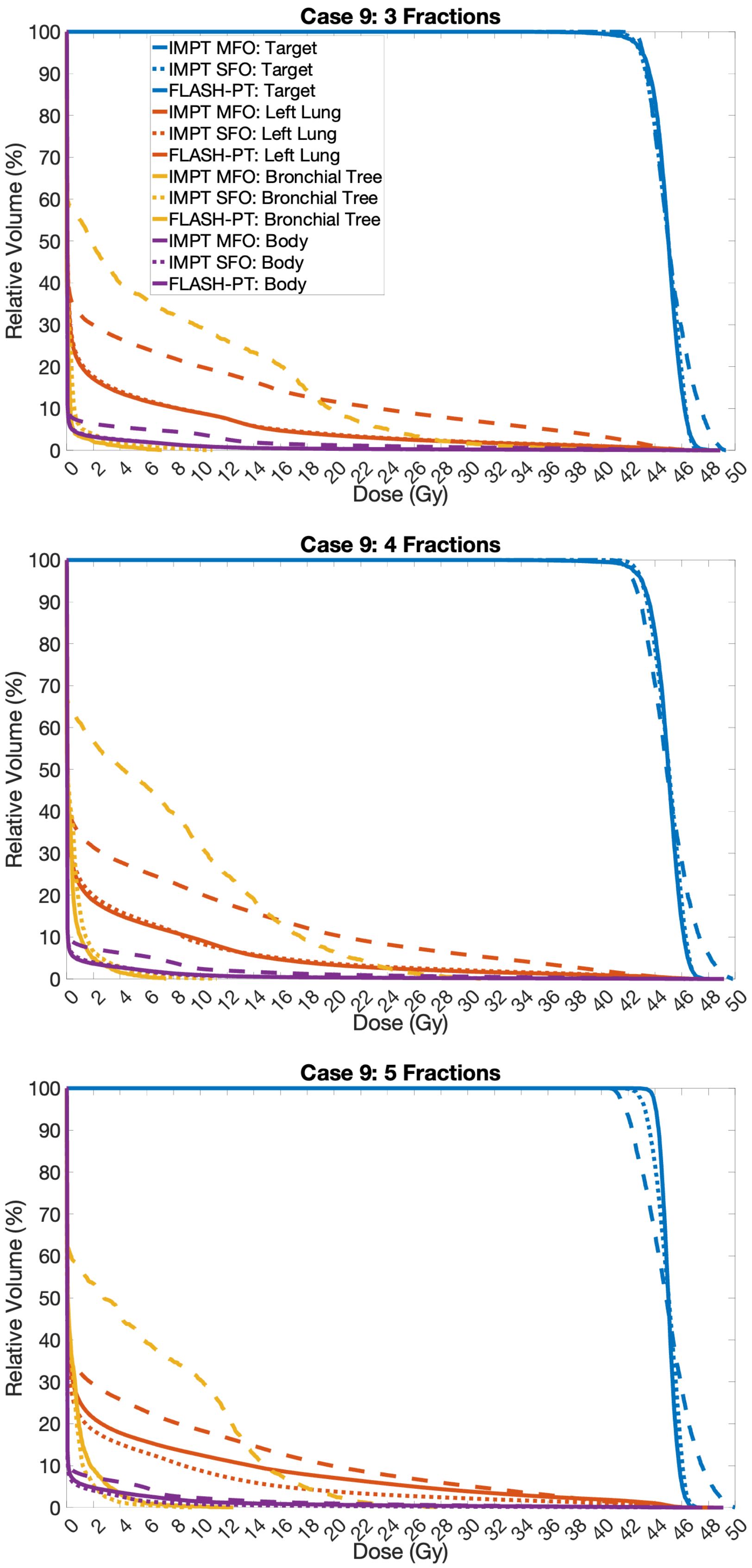

Figure S3: Dose volume histograms for Case 9. In each case, doses to the target and OAR structures for each treatment technique (IMPT MFO, IMPT SFO, and FLASH-PT), and for each fractionation scheme, are displayed.

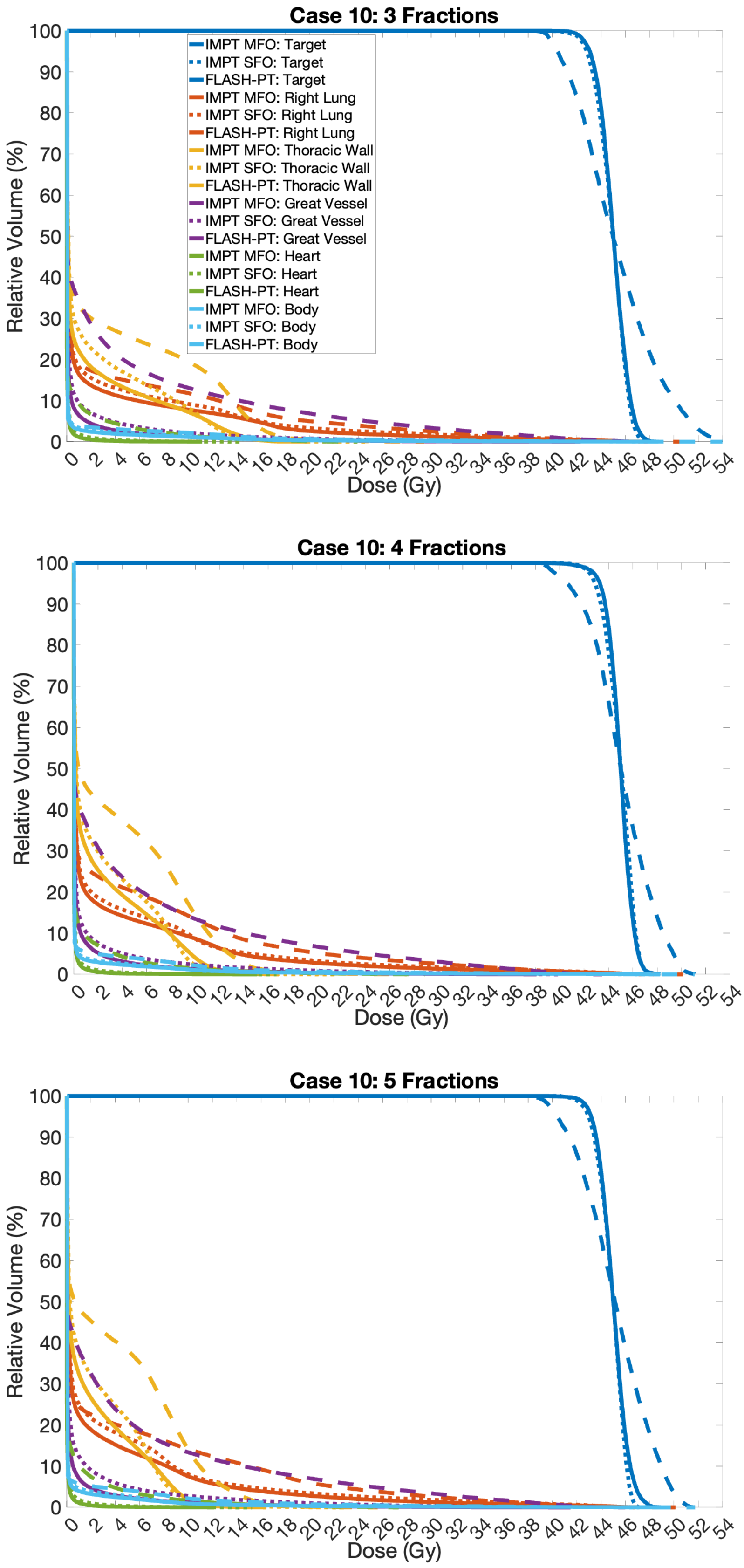

Figure S4: Dose volume histograms for Case 10. In each case, doses to the target and OAR structures for each treatment technique (IMPT MFO, IMPT SFO, and FLASH-PT), and for each fractionation scheme, are displayed.

Table S1: Table of spot spacings (mm), layer spacings (mm), and target margins for spot placements (mm) for each patient case when generating treatment plans using standard IMPT (MFO).

**Patient Cases**	Spot Spacing (mm)	Layer Spacing (mm)	Target Margin (mm)
5	2	2	[2,2,2]
6	3	3	[2,2,2]
7	2	2	[4,4,4]
8 (3 fractions)	3	3	[5,5,5]
8 (4 fractions)	3	3	[5,5,5]
8 (5 fractions)	3	3	[5,5,5]
9 (3 fractions)	3	3	[5,5,5]
9 (4 fractions)	4	4	[5,5,5]
9 (5 fractions)	3	3	[5,5,5]
10 (3 fractions)	3	3	[5,5,5]
10 (4 fractions)	3	3	[5,5,5]
10 (5 fractions)	3	3	[5,5,5]

Table S2: Table of spot spacings (mm), layer spacings (mm), and target margins for spot placement (mm) for each patient case and each fraction number when generating IMPT plans using one beam per fraction (SFO).

**Patient Cases**	Fraction Number	Spot Spacing (mm)	Layer Spacing (mm)	Target Margin (mm)
5	1	1	1	[2,2,2]
	2	1	1	[2,2,2]
	3	1	1	[2,2,2]
6	1	1	1	[2,2,2]
	2	1	1	[3,3,3]
	3	1	1	[3,3,3]
7	1	2	2	[5,5,5]
	1	2	2	[5,5,5]
8 (3 fractions)	1	2	2	[5,5,5]

	2	5	5	[5,5,5]
	3	5	5	[5,5,5]
8 (4 fractions)	1	5	5	[5,5,5]
	2	5	5	[5,5,5]
	3	5	5	[5,5,5]
	4	5	5	[5,5,5]
8 (5 fractions)	1	5	5	[5,5,5]
	2	5	5	[5,5,5]
	3	5	5	[5,5,5]
	4	5	5	[5,5,5]
	5	5	5	[5,5,5]
9 (3 fractions)	1	2	2	[5,5,5]
	2	2	2	[5,5,5]
	3	2	2	[5,5,5]
9 (4 fractions)	1	2	2	[5,5,5]
	2	2	2	[5,5,5]
	3	2	2	[5,5,5]
	4	2	2	[5,5,5]
9 (5 fractions)	1	2	2	[5,5,5]
	2	2	2	[5,5,5]
	3	2	2	[5,5,5]
	4	2	2	[5,5,5]
	5	2	2	[5,5,5]
10 (3 fractions)	1	3	3	[5,5,5]
	2	5	5	[5,5,5]
	3	3	3	[5,5,5]
10 (4 fractions)	1	3	3	[5,5,5]
	2	5	5	[5,5,5]
	3	5	5	[5,5,5]
	4	2	2	[5,5,5]

10 (5 fractions)	1	3	3	[5,5,5]
	2	5	5	[5,5,5]
	3	5	5	[5,5,5]
	4	3	3	[5,5,5]
	5	3	3	[5,5,5]

Table S3: Table of spot spacings (mm), layer spacings (mm), target margins for spot placement (mm), and aperture margins (mm) for each patient case and each fraction number when generating Bragg peak FLASH-PT treatment plans using one beam per fraction (SFO).

**Patient Cases**	Fraction Number	Spot Spacing (mm)	Layer Spacing (mm)	Target Margin (mm)	Aperture Margin (mm)
5	1	8	13	[4,4,4]	[4,4,4]
	2	9	16	[12,12,12]	[5,5,5]
	3	9	19	[12,12,12]	[5,5,5]
6	1	7	14	[5,5,5]	[4,4,4]
	2	7	18	[5,5,5]	[4,4,4]
	3	7	18	[8,8,8]	[3,3,3]
7	1	10	18	[12,12,12]	[12,12,12]
	1	12	18	[14,14,14]	[14,14,14]
8 (3 fractions)	1	10	10	[18,18,18]	[15,15,15]
	2	10	25	[12,12,12]	[12,12,12]
	3	18	18	[15,15,15]	[15,15,15]
8 (4 fractions)	1	15	20	[12,10,12]	[12,12,12]
	2	15	15	[12,12,12]	[12,12,12]
	3	18	15	[15,15,15]	[15,15,15]
	4	18	15	[12,12,12]	[12,12,12]
8 (5 fractions)	1	10	25	[12,10,12]	[12,12,12]
	2	10	25	[12,10,12]	[12,12,12]
	3	18	20	[15,15,15]	[15,15,15]
	4	10	14	[12,10,12]	[12,12,12]
	5	9	18	[12,10,12]	[12,12,12]
9 (3 fractions)	1	14	20	[18,18,18]	[15,15,15]
	2	10	20	[18,18,18]	[15,15,15]

	3	10	20	[18,18,18]	[18,18,18]
9 (4 fractions)	1	10	20	[15,15,15]	[15,15,15]
	2	14	18	[18,18,18]	[15,15,15]
	3	14	18	[18,18,18]	[15,15,15]
	4	10	20	[18,18,18]	[15,15,15]
9 (5 fractions)	1	8	18	[12,10,12]	[12,12,12]
	2	18	20	[18,18,18]	[15,15,15]
	3	12	18	[18,18,18]	[18,18,18]
	4	15	18	[18,18,18]	[15,15,15]
	5	12	18	[12,10,12]	[12,12,12]
10 (3 fractions)	1	12	25	[15,15,15]	[12,12,12]
	2	10	20	[15,15,15]	[15,15,15]
	3	12	25	[18,18,18]	[15,15,15]
10 (4 fractions)	1	15	18	[18,18,18]	[18,18,18]
	2	15	12	[16,16,16]	[16,16,16]
	3	13	10	[18,18,18]	[18,18,18]
	4	13	20	[18,18,18]	[15,15,15]
10 (5 fractions)	1	12	20	[18,18,18]	[18,18,18]
	2	13	15	[15,15,15]	[15,15,15]
	3	13	15	[18,18,18]	[18,18,18]
	4	13	15	[18,18,18]	[18,18,18]
	5	13	20	[18,18,18]	[14,14,14]
